# Advances in Detecting Cystic Echinococcosis in Intermediate Hosts and New Diagnostic Tools: A Literature Review

**DOI:** 10.3390/vetsci11060227

**Published:** 2024-05-21

**Authors:** Ashkan Hajjafari, Soheil Sadr, Cinzia Santucciu, Giovanna Masala, Mansour Bayat, Narges Lotfalizadeh, Hassan Borji, Soroush Partovi Moghaddam, Khashayar Hajjafari

**Affiliations:** 1 Department of Pathobiology, Faculty of Veterinary Specialized Science, Science, and Research Branch, Islamic Azad University, Tehran 1477893855, Iran; hajjafari.2014@gmail.com (A.H.); soroush.partovi99@gmail.com (S.P.M.); 2 Department of Pathobiology, Faculty of Veterinary Medicine, Ferdowsi University of Mashhad, Mashhad 917794897, Iran; soheil.sadr42@gmail.com (S.S.); narges.lotfalizadeh@alumni.um.ac.ir (N.L.); 3 WOAH and National Reference Laboratories for Echinococcosis, Animal Health, Istituto Zooprofilattico Sperimentale della Sardegna, 07100 Sassari, Italy; giovanna.masala@izs-sardegna.it; 4 Medical Graduated Student, Medical School, Shahid Bahonar University of Medical Sciences, Kerman 7618411764, Iran; hajjafari.kh@gmail.com

**Keywords:** diagnosis, *Echinococcus granulosus*, hydatid cyst, intermediate host, nanobiosensors

## Abstract

**Simple Summary:**

Despite its importance to global health, hydatid disease remains difficult to diagnose and control without accurate and accessible diagnostic tools. A One Health approach is necessary for cystic echinococcosis (CE), a zoonotic disease affecting humans and animals. Antibodies detection of *Echinococcus granulosus* by Enzyme-Linked Immunosorbent Assay (ELISA) and immunoblotting can confirm CE diagnosis, particularly in cases where other techniques may fail. A correct and early diagnosis is fundamental to determining the treatment outcome of the CE patient. Nanotechnologies and nanobiosensors have advanced diagnostic capabilities in recent years. The development of nanobiosensors has the potential to bridge the gap between human and veterinary diagnostics, enabling more integrated surveillance and control strategies. Nanobiosensors present a significant step forward in CE detection in resource-constrained settings when integrated into field-based surveillance systems. Due to their portability and ease of use, these sensors can be used as point-of-care diagnostic devices, enabling timely, decentralized diagnosis. Ensuring access to advanced laboratory tools in regions with limited access is crucial.

**Abstract:**

Cystic echinococcosis (CE) is a zoonotic disease affecting humans and animals. Despite a lack of clarity about many details of parasite–intermediate host interactions, the nature of the immune responses triggered by hydatid infection has revealed new perspectives. This study discusses the latest advances in elucidating the immunologic mechanism of echinococcosis and its detection and potential approaches to enhance serodiagnosis accuracy. Moreover, nanobiosensors have been evaluated according to their potential to improve treatment efficiency and aid in an early diagnosis of cystic echinococcosis. The serum of an intermediate host can diagnose CE by analyzing antibodies induced by *Echinococcus granulosus*. Among the most notable features of this method are its noninvasive ability and high sensitivity, both of which make it an excellent tool for clinical diagnosis. Several serological tests, including ELISAs and immunoblotting, can detect these antibodies to assess the disease’s state and determine the treatment outcome. A thorough understanding of what cross-reactivity means and the stage of the disease are crucial to interpreting serological results. Nanobiosensors have also proven better than conventional biosensors in detecting hydatid cysts. Additionally, they are highly sensitive and versatile when detecting specific biomarkers, improving diagnostic accuracy. These immunomodulatory molecules, induced by *E. granulosus*, are a good candidate for diagnosing cystic echinococcosis because they alter intermediate host immune responses. Hydatid cyst detection is also enhanced through nanobiosensors, which provide better accuracy.

## 1. Introduction

*Echinococcus granulosus sensu lato* (*s.l.*) is a member of the Taeniidae family, a group of cestode parasites relevant in the medical and veterinary fields since it is the etiologic agent of cystic echinococcosis (CE) [1]. It is a parasite that belongs to the same family of *Taenia solium* and *Taenia saginata*. This parasite is widespread worldwide, except in Antarctica, New Zealand, and Iceland [2]. There is genetic diversity within *E. granulosus s.l.*, which contributes to its complexity. In terms of taxonomy, this species consists of several distinct genotypes or strains [3]. Several genotypes of *E. granulosus s.l.* have adapted to different host species [4]. Generally, genotypes G1 through G10 are geographically associated with specific intermediate hosts and areas [5,6]. Moreover, these genotypes are further subdivided into sub-genotypes [7].

The species *E. granulosus s.l.* has now been subdivided into *E. granulosus sensu stricto* (*s.s.*) (including the genotypes G1 and G3; sheep and buffalo strains), *E. equinus* (G4; horse strain), and *E. ortleppi* (G5; cattle strain), according to structural, molecular, and ecological aspects [7]. There is still some uncertainty regarding the species status of the remaining genotypes, G6 (camel strain), G7 (pig strain), G8, and G10 (cervid strain) [8]. Previously, Nakao et al., [9,10,11] proposed that these genotypes should be classified as a single species (*E. canadensis*), while Thompson suggested a separate taxon for domestic strains (G6 and G7; *E. intermedius*), sylvatic genotypes (G8 and G10; *E. canadensis*) and *E. felidis* [12]. Parasitology and epidemiology rely primarily on mitochondrial genes and the *ITS1* region for genotyping *E. granulosus s.l.* [13]. By employing genotyping methods, scientists can learn more about the species’ diversity, transmission, and population structure. 

**Figure 1:**
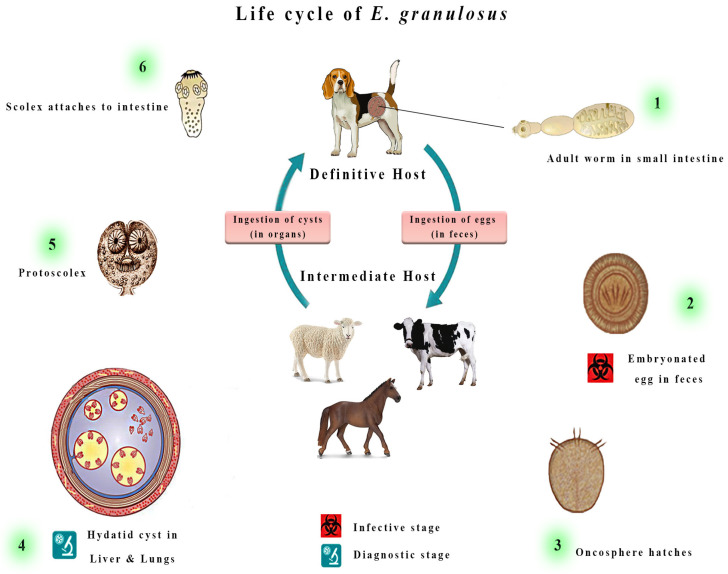
The adult form of *Echinococcus granulosus sensu lato* (*s.l.*) is typically 2–7 mm long and resides in the small intestine of its definitive host. Gravid proglottids release eggs, which are then excreted in the feces and are infectious. The eggs hatch in the small intestine upon ingestion by suitable intermediate hosts, releasing six-hooked oncospheres. These oncospheres penetrate the intestinal wall and migrate through the circulatory system, eventually reaching various organs, particularly the liver and lungs. Once in these organs, the oncosphere develops into a thick-walled hydatid cyst, which gradually enlarges, producing protoscolices and daughter cysts that fill the cyst interior. The definitive host becomes infected by ingesting the cyst-containing organs of the infected intermediate host. After ingestion, the protoscolices evaginate, attach to the intestinal mucosa, and develop into adult stages within 32 to 80 days.

There are two kinds of hosts in the life cycle of *E. granulosus s.l.* (Figure 1) [14]. Specifically, it uses canids like domestic dogs as definitive hosts [15]. Upon reaching adulthood, Echinococcus worms shed their eggs into the feces that, if released into the environment, may be ingested with contaminated food by intermediate hosts, represented by sheep and other ruminants such as cattle, sheep, and goats [16]. Humans are accidental dead-end intermediate hosts [17]. 

**Figure 2:**
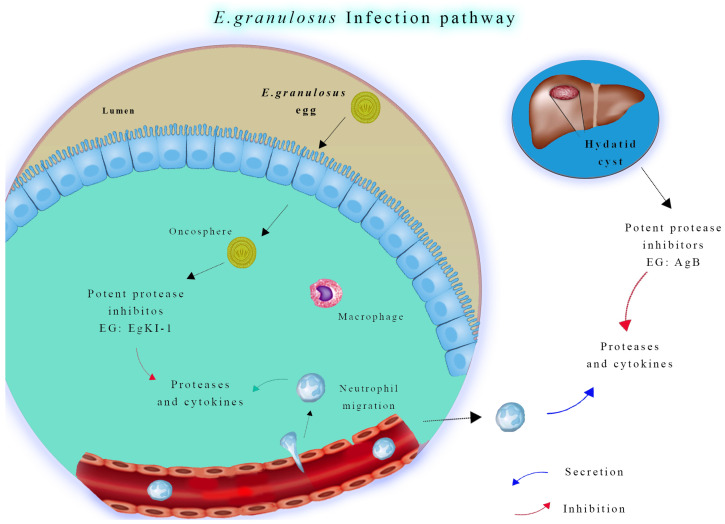
Neutrophil function in *Echinococcus granulosus* infection. During the early stage of infection, neutrophils and macrophages migrate to the intestinal mucosa to combat invading oncospheres. In later stages, if hydatid cyst fluid leaks from a ruptured cyst, neutrophils are attracted to the area. However, antigen B inhibits neutrophil chemotaxis and neutrophil elastase, which helps protect protoscoleces, allowing them to develop into new cysts.

These eggs will hatch into the bowel (Figure 2) and release tiny embryos termed oncospheres, which, once ingested, penetrate the intestinal wall of the intermediate hosts and migrate across bloodstreams to various tissues, with preference given to the liver and lungs [18]. This process results in the formation of cystic lesions, which can fill with fluid and grow to extremely large sizes that damage the host’s organs, resulting in crippling symptoms and even death in some cases [19].

Aside from the detrimental effects of this damage on individual animals, it also has substantial economic consequences for the global livestock industry [20]. A significant economic impact is associated with CE in ruminant populations [21]. It reduces milk and meat production in cattle, resulting in a decline in productivity, causing the slaughter or culling of infected animals, and directly affecting the meat and dairy industries [20]. There are substantial public health implications associated with echinococcosis, with millions of people at risk of contracting the disease from contaminated food and water, and a very high economic burden [22]. Between 1997 and 2021, a systematic review identified 64,745 cases throughout 40 European countries. Southeastern European countries were the epicenter of southeastern European epidemics, with an average incidence rate of 0.64 cases per 100,000 people in Europe [23]. Robust and efficient diagnostic approaches are required to diagnose echinococcosis accurately because of its insidious nature. A timely diagnosis is essential for managing the disease effectively and preventing its spread [24]. Diagnosing accurately enables healthcare professionals to intervene promptly, reducing the impact of CE on human and animal populations [25].

Cystic structures can be mainly diagnosed using ultrasounds (US), primarily for abdominal involvement, and conventional chest radiography [26]. At the same time, Computed Tomography (CT) and Magnetic Resonance Imaging (MRI) are imaging techniques that are useful in confirming previous exams and giving deeper details on organ structures that are usually required before surgery [27]. Moreover, CE cysts can be studied in the US according to the World Health Organization and Informal Working Group on Echinococcosis (WHO-IWGE) classification guidelines [28]. In detail, lesions are categorized into different stages, including active stages CE1 and CE2, transitional stage CE3 (CE3a and CE3b), and finally, the inactive group comprising CE4 and CE5 [29]. 

The enzyme-linked immunosorbent assay (ELISA) and immunoblotting (IB) have proven valuable methods [30]. These tests aim to detect antibodies specific to antigens of *E. granulosus s.l* that are produced by the host’s immune response. Imaging techniques and serological tests are reliable tools for diagnosing *E. granulosus s.l.* infections. In some cases, however, the immunodiagnostic tests have shown limitations in their sensitivity and specificity in accurately detecting Echinococcus-specific antibodies [30,31,32]; they often are not able to detect an antibody titer at the early stage (CE1) or late stage (CE4 or CE5) of an echinococcal cyst and in subjects with cysts in organs other than the liver [33,34,35,36]. 

With the advent of nanotechnology, the diagnostic field has been revolutionized. Nanobiosensors, which can detect infectious agents like echinococcosis, are among the most promising developments in this field, and recently, there was an attempt to develop nanobiosensors for schistosomiasis [37]. Nanobiosensors are innovative devices that combine nanomaterials with biological molecules to detect biomarkers with excellent sensitivity and precision so that biomarkers can be identified with high sensitivity and accuracy [38,39]. The remarkable versatility and efficiency of nanobiosensors distinguish them from conventional diagnostic tools [40]. In addition to rapid results, these devices are often more economical for diagnostic purposes. Moreover, nanobiosensors can detect multiple analytes simultaneously, making them suitable for detecting complex diseases like echinococcosis [41]. Researchers need to explore the potential for nanobiosensors to revolutionize the detection and management of echinococcosis. Through nanotechnology, these sensors detect genetic markers or *E. granulosus* antigens with unprecedented accuracy. Therefore, they improve diagnostic reliability by reducing false positives and negatives [42]. Biosensors can be deployed in the field, allowing for rapid and onsite diagnosis, which is vital in providing on-time care [43]. Specifically, this comprehensive review provides an update and in-depth evaluation of testing for intermediate host CE and explores the future of diagnostic methodologies incorporating nanobiosensors.

## 2. Methods for Diagnosis of Cystic Echinococcosis in Intermediate Hosts

**Figure 3:**
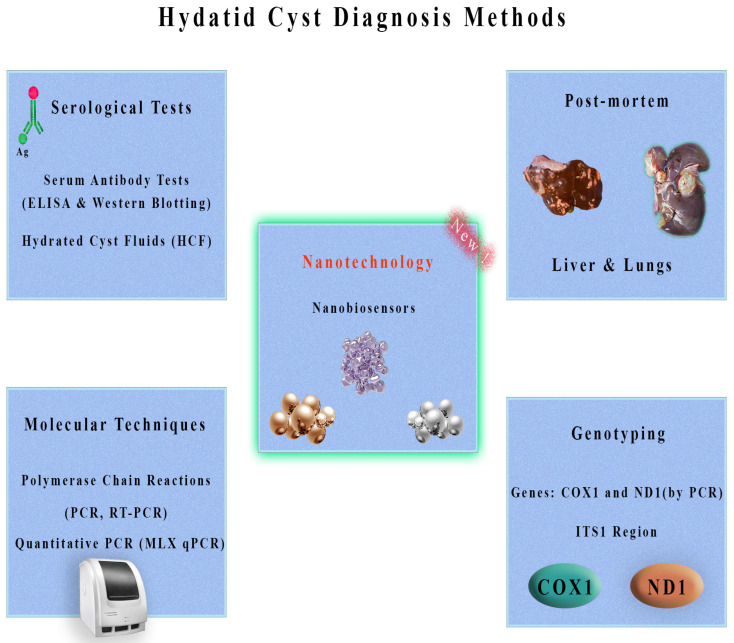
Overview of hydatid cyst diagnosis methods. Serological tests, including serum antibody tests (ELISA and Western blotting) and examination of hydatid cyst fluids (HCF), are commonly used diagnostic tools. Molecular techniques such as polymerase chain reactions (PCR, RT-PCR) and quantitative PCR (MLX qPCR) offer high sensitivity and specificity. Post-mortem examination of the liver and lungs can also aid in diagnosis. Genotyping methods targeting genes, such as *COX1* and *ND1* (by PCR) and the *ITS1* region, provide valuable information. Emerging technologies, like nanobiosensors using materials such as gold, silver, and others, show promise for future diagnostic advancements.

Different methods and techniques, as shown in Figure 3, facilitate the detection of CE in intermediate hosts, each of which plays a unique and harmonious role [44]. Human and animal detection methods can be combined to provide a comprehensive view of the condition prevalence in a population [45]. Using this collective insight, researchers hope for effective management and control strategies. Combining these diagnostic methods with an interdisciplinary approach is the key to combating this insidious zoonotic disease.

### 2.1. Serological Tests

Public and animal health surveillance rely on CE diagnosis on serological assays to support image techniques. These tests, particularly ELISAs and IB, can detect *E. granulosus* antibodies [46]. In large-scale surveillance efforts, ELISA is often used to detect specific antibodies, which help estimate infection prevalence and identify asymptomatic patients [30]. While IB is complex and more expensive, it is usually used as a valuable confirmation test, enhancing diagnostic specificity [47]. In epidemiological studies, the serological test is crucial for quantifying infection prevalence and detecting subclinical infections [48]. ELISA methods can diagnose CE in intermediate hosts like sheep and cattle functionalized with *E. granulosus* Antigen B (AgB) [49]. ELISAs with sheep conjugates and gold nanoparticles with anti-sheep conjugates have been used [50]. Both methods were highly sensitive (92% and 100%) and specific (96% and 96%) for sheep. Antigen B ELISAs with gold nanoparticles improved specificity and sensitivity, especially when gold nanoparticles were incorporated into the design [51].

Alternatively, an IgG polyclonal antigen-based ELISA was developed to detect circulatory *E. granulosus* antigens in camels affected with CE, using hydatid cyst germinal layer antigen (GlAg) [46]. A specific antibody polyclonal antigen sandwich ELISA method was developed to detect *E. granulosus* antigens circulating in camels with hydatid cysts before slaughtering, and its applicability to serodiagnosis of cystic echinococcosis in animals was also examined. According to postmortem examination of slaughtered camels, 46.5% had hydatid cysts, which was confirmed by molecular identification. Hepatic echinococcosis was much less common than pulmonary echinococcosis. Isolated hydatid cyst germinal layer antigen was used to raise IgG polyclonal antibodies, demonstrating high sensitivity (98.9%) and specificity (94.9%). However, it was found that *Fasciola gigantica* and Myiasis (*Cephalopina titillator* larvae) had minimal cross-reactivity with the assay, indicating its specificity and reliability. Compared with a postmortem inspection (46.5%), the Sandwich ELISA detected 48.7% cystic echinococcosis in camel serum. It shows promise as an early detection and treatment technique for camel echinococcosis, with significance for both human and veterinary applications.

Various intermediate hosts were assessed for efficacy as diagnostic antigens for cystic echinococcosis using hydatid cyst fluids (HCF) [52]. ELISA tests were performed on sixteen crude HCF samples to determine whether they reacted with human serum. Variations in reactivity were found among HCF samples, regardless of protein content. Different protein bands were identified by SDS-PAGE, including a 64 KDa protein that may be useful for diagnosing human cystic echinococcosis. In addition, indirect ELISA was used to diagnose cystic echinococcosis using recombinant B-EpC1 fusion antigens. Recombinant antigen results showed 95.2% and 96.8% diagnostic sensitivity and specificity. According to the study, human cystic echinococcosis can be detected using a recombinant fusion antigen for specific anti-hydatid cyst antibodies.

A Bayesian Latent Class Analysis (LCA) model was employed to estimate the incidence of cystic echinococcosis in sheep samples from Argentina’s Río Negro province, considering diagnostic uncertainties [53]. The indirect ELISA rEgAgB8/2 was assessed to detect *E. granulosus* in sheep. An optimal optical density threshold demonstrated 55% and 68% effective sensitivity and specificity in the sampled population. Using the ELISA, there was an 80% probability of correctly classifying infection at the flock level. These findings support using ELISAs for flock-level cystic echinococcosis surveillance in the region, complementing human health efforts and reinforcing One Health initiatives. Certainly, serological tests have been used most efficiently in humans, as there is less chance of cross-reaction with antigens or antibodies derived from other similar parasites. 

### 2.2. Post-Mortem Inspection

Postmortem analyses are comprehensive examinations presenting high reliability for CE diagnosis in slaughterhouses or necropsies [54]. Firstly, all organs, including the liver and lungs, are meticulously examined by trained inspectors by visually identifying characteristic cysts. An overview of cyst size, number, and location provides invaluable insights into disease characterization, prevalence estimation, and epidemiological studies [55]. Postmortem inspections prevent the entry of infected meat into the food supply chain, safeguarding human health by preventing the spread of disease [56]. However, this method is limited by its inherent limitations, primarily its posthumous nature, and it cannot detect or treat disease early or intervene immediately [57]. Several hydatid cysts were found during a postmortem examination of a cow with severe dyspnea [58]. There were several hydatid cysts in the lungs, including one near the bifurcation of the trachea. Postmortem diagnosis was crucial to understanding the impact of CE on livestock in this case.

After post-mortem inspection, the CE diagnosis can be confirmed following a direct analysis of cystic liquid using microscopy, which can detect the presence of protoscoleces or their part, the hooks, and their viability. *E. granulosus* may cause hydatid disease in many warm-blooded animals, including pigs [59]. After postmortem examination, if cysts are not visible with the naked eye because of their small size, it is possible to examine organs, such as the liver, by histopathology [60]. These techniques lead to evidence of pathognomonic features typical of *E. granulosus* infection characterized by different layers: the granulomatous reaction surrounding the parasitic structure host-produced, one thick laminated and acellular, another cellular germinal layer, and finally, the brood capsules containing protoscolices [61]. A sow slaughtered because of progressive weakness was found to have multiple vesicular lesions by histology [62]. This finding emphasizes the zoonotic nature of the disease and its potential to spread to livestock and humans from rural dogs used to protect sheep farms. Transmission is more likely since regular anthelmintic treatment is not provided in such settings. These insights are essential to designing regional control strategies to reduce hydatid disease prevalence in livestock and, therefore, human infections [48].

### 2.3. Molecular Techniques

Polymerase Chain Reactions (PCR) have revolutionized the precision and efficacy of all diagnostic fields [63]. In particular, in several parasites such as *E. granulosus*, the possibility of detecting and identifying the DNA allows for making a clear diagnosis where other techniques have failed for several reasons since PCR offers unparalleled capabilities [64]. The molecular approach provides diagnosticians with the advantage of confirming the presence of the parasite and, most importantly, identifying its species and genotype or strain [65]. Scientific research, epidemiological investigations, and early disease detection rely on molecular techniques, especially PCR, known for their sensitivity and specificity [66]. Additionally, these techniques enable us to understand genetic variability and geographic distribution so that specific interventions can be tailored to particular regions [67]. A single-tube nested PCR (STNPCR), designed to detect the *COI* gene, was evaluated for its efficacy as an indicator of *Echinococcus* spp. DNA. STNPCR displayed 100 times increased sensitivity compared to conventional PCR, making it suitable for gene sequencing and epidemiological investigations [68]. Genetic diversity and evolution can be studied through the ability of *Echinococcus* spp. to amplify small amounts of genomic DNA [67].

A PCR assay detected specific cell-free DNA (cfDNA) from *E. granulosus s.l.* in the sera of naturally infected sheep [69]. Researchers evaluated the modified phenol–chloroform method for preserving cfDNA and found that increased serum volume and template DNA enhanced sensitivity. PCR amplicons were sequenced to confirm the results. With larger volumes of serum and DNA template and a semi-nested PCR protocol, sensitivity increased to 95%, offering hope for early diagnosis of echinococcosis. An epidemiological investigation was conducted in the Qinghai-Tibetan Plateau area of *E. multilocularis*, *E. granulosus s.s*., and *E. shiquicus* coinfections. To detect these *Echinococcus* species simultaneously, a triplex TaqMan-minor groove binding probe was developed from canid feces for real-time polymerase chain reactions (RT-PCR). Because it is highly specific, precise, and stable, this triplex RT-PCR assay can be used for epidemiological investigations of echinococcosis [70]. 

A consensus has yet to be reached regarding the organ preferences of currently known species and subspecies. An analysis of potential organ distribution patterns of 89,359 *Echinococcus* cysts from 47 different countries’ intermediate hosts was undertaken about genotypes/species, utilizing statistical methods and following PRISMA guidelines [71]. There was a significant increase in *E. granulosus s.s.* (G1, G3) and *E. canadensis* (G7) in sheep’s and pigs’ livers. In contrast, *E. ortleppi* and *E. canadensis* G6 were significantly higher in cattle and camels’ lungs. It is necessary to investigate whether *Echinococcus* displays organ tropism through species/genotype or host dependence in the future. Cystic echinococcosis may be diagnosed and treated more precisely based on organ-specific characteristics if additional research can provide significant insight. 

Using a molecular screening approach, *Echinococcus* spp. and other tapeworms were detected in fecal samples collected from wild carnivores in central Italy [8]. PCR targeted diagnostic DNA fragments from the *nad1*, *rrnS*, and *nad5* genes. Other tapeworms were more frequently detected than *E. granulosus s.s*. (genotype G3), including *Mesocestoides* spp. and *Taenia* spp. *Echinococcus granulosus s.s*. was less prevalent in wild carnivores in this region, underscoring the need for passive surveillance. 

#### 2.3.1. Genotyping with Mitochondrial Genes

Parasitology and epidemiology require mitochondrial gene genotyping of *E. granulosus s.l.* [72]. Studies of parasite diversity and population structure using mitochondrial genes are ideal. For an assessment of *Echinococcus* spp. prevalence and transmission dynamics, it is necessary to collect samples from humans and potentially infected animals. To understand the extent of the parasite’s spread and the potential risks to human health, biological samples are collected from both human populations and animals at risk of infection [16]. Preservation and handling of these samples are essential for preventing DNA degradation [73]. Analyzing the parasite’s DNA requires an extraction step for isolation. Due to their diversity, specific mitochondrial genes, including *COX1* and *ND1*, are targeted for genetic studies [74]. PCR is used to amplify mitochondrial genes and increase the quantity of genetic material in mitochondria [72]. Genetic sequences of mitochondria are determined using DNA sequencing technologies like Sanger and Next-Generation Sequencing (NGS) [75]. Genetic variations associated with geographic regions, hosts, and transmission pathways can be revealed through bioinformatic analysis of mitochondrial haplotypes or genotypes within the samples [76]. The phylogenetic analysis of mitochondrial genes is an effective tool for understanding the evolution of genotypes. Besides revealing genetic relationships, it is possible to build a phylogenetic tree useful to distinguish genotypes and show patterns of infection and transmission [67]. Genotyping mitochondrial genes is a significant component of epidemiological research. An effective control measure is determined by identifying sources of infection as well as understanding transmission dynamics [77]. Cystic echinococcosis can be prevented and treated, reducing the burden on humans and animals.

#### 2.3.2. Genotyping with the *ITS1* Region

Genotyping *E. granulosus s.l.* based on the *ITS1* region, located within ribosomal DNA, is very useful due to its high genetic variability [78]. This region is used to understand parasite genetic makeup. Cysts must be collected from an infected host first. Subsequently, cystic membranes or protoscoleces can be collected. Amplification of parasite DNA with PCR involves targeting the highly variable *ITS1* region using specific primers [79]. DNA sequencing technology, such as Sanger sequencing or NGS, is also used to decode the genetic code of the *ITS1* region [80]. Genetic data is analyzed using bioinformatics tools and databases to identify genotypes and haplotypes. An evolutionary tree is constructed by phylogenetic analysis based on the genetic data of *E. granulosus s.l*. [81]. Infection sources and the parasite’s spread can be tracked using this information. Epidemiological studies can benefit from genotyping and haplotyping data. Different regions and hosts have additional genetic diversity, which can be used to assess risk factors, identify infection sources, and analyze transmission dynamics [82]. Improving the knowledge and understanding of this zoonotic disease in depth is essential to preventing and treating it.

### 2.4. Nanobiosensors for Improved Hydatid Cyst Detection 

**Figure 4:**
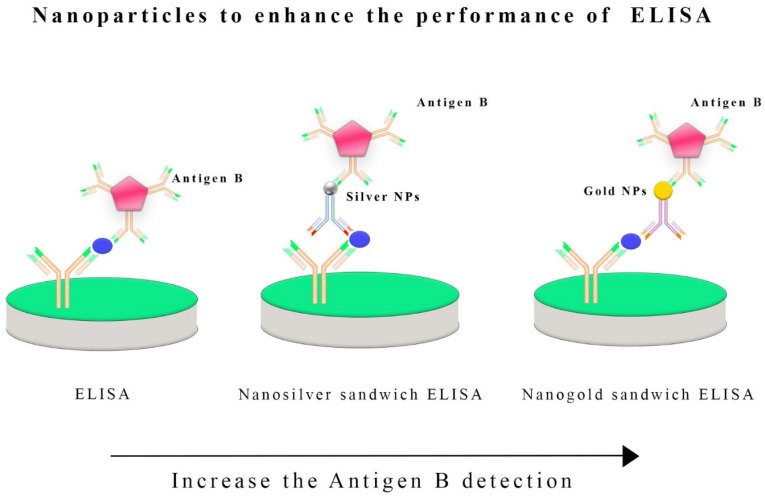
Nanoparticles, such as silver or gold nanoparticles, have been integrated into the enzyme-linked immunosorbent assay (ELISA) to boost its sensitivity and detection capabilities. Incorporating these nanoparticles into the ELISA process enhances the detection of specific antigens, such as antigen B, in *E. granulosus* infections. These nanoparticles act as carriers for antibodies or antigens, increasing the surface area available for binding and allowing for more efficient interactions between the target antigen and the detection antibodies. The use of silver and gold nanoparticles also leverages their unique optical properties, which can improve the signal detection methods employed in ELISA, ultimately enhancing its overall sensitivity and performance in diagnosing infections caused by *E. granulosus* and potentially other pathogens.

Detecting the zoonotic agents of CE with nanobiosensors represents an exciting frontier in disease diagnosis [25]. Indeed, these innovative tools can be employed on animals and humans, and this parasitic cestode, *E. granulosus s.l.*, may induce CE, a multifaceted zoonotic illness [83]. Several reports have described enhanced performance, such as sensitivity, specificity, speed, adaptability, and portability, through the convergence of nanotechnology and biosensing technology [84]. The zoonotic nature of CE requires the development of a sensitive method for diagnosing the disease, particularly in cases where the other techniques present a low capability [85]. *E. granulosus s.s.* G1-G3 causes human hydatid cysts, which are prevalent almost worldwide [86]. Several surveys indicate that this parasite causes significant economic losses to all intermediate hosts [87]. In a study by Shirazi et al. (2022) [51], serum samples were collected from newborns as negative controls and sheep with CE as positive controls. A specific ELISA technique was used to detect CE in sheep using an Iranian native B antigen. The first method used anti-sheep conjugate (SIGMA, Darmstadt, Germany, at 1:3000 dilution), and the second method used gold nanoparticles in combination with anti-sheep conjugate. Combining ELISA and nanoparticles has enhanced the detection efficacy (Figure 4). AgB-ELISAs were 92% sensitive and 96% specific in sheep. In comparison, Nano-ELISAs with gold nanoparticles were 100% sensitive and 96% specific. When gold nanoparticles are conjugated with anti-sheep antibodies in an ELISA design, specificity and sensitivity will increase significantly, especially at low titers [51].

Nanoparticles of gold (AuNPs) were synthesized by Jafari et al. (2022) [88] to focus on developing a highly sensitive nanobiosensor for diagnosing hydatid cysts in intermediate hosts. ELISA-based techniques and Tetramethylbenzidine (TMB) were used to test IgG antibodies against *E. granulosus* antigen coated on microwells. Spectrophotometry was used to measure the absorption rate of AuNPs synthesized with TMB. The Nanobiosensor can detect *E. granulosus* antibodies with detection limits as low as 0.001 g per milliliter. The results confirmed that the designed nanobiosensor was completely specific for detecting *E. granulosus* antibodies.

The study by El-Sherbini et al. (2022) describes how AuNP was used in a genomic microarray to precisely identify the *COX1* mitochondrial gene in Echinococcus strains [89]. This innovative approach eliminates DNA amplification, making it a cost-effective alternative for laboratories with limited equipment. Microarray analysis was performed on 30 human hydatid cyst samples. These specimens were analyzed using amino-labeled probes corresponding to 10 genotypes of *E. granulosus s.l.* Results showed a high prevalence of camel strain G6 in 63.3% of cases of human CE, while the G1 genotype comprised 36.7%. The G6 genotype is associated with positive serological results and multiple organ involvement.

**Figure 5:**
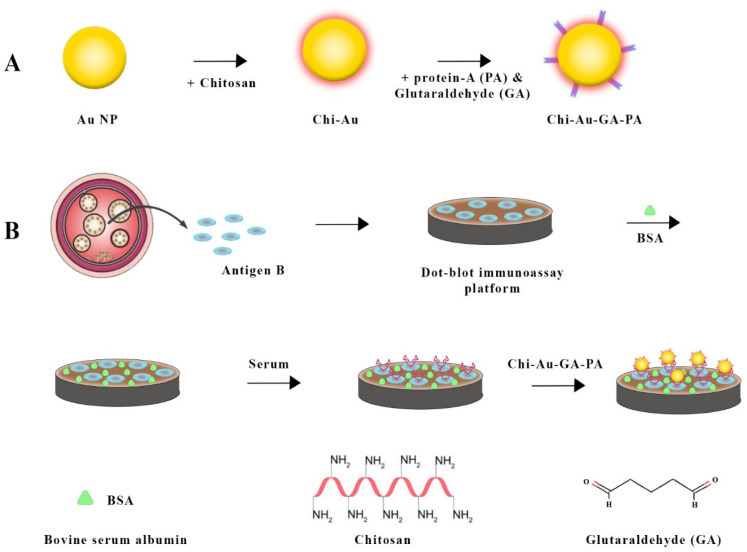
Combining chitosan–gold nanoparticles in a sandwich-based biosensor for *Echinococcus granulosus* detection involved several steps. (**A**) gold nanoparticles were synthesized using chitosan. Next, the chitosan–gold nanoparticle surface was activated using glutaraldehyde (GA) and then conjugated with Protein A. In the next step (**B**), hydatid cyst antigen (Ag B) was immobilized on a nitrocellulose (NC) membrane, which was then blocked with bovine serum albumin (BSA). The membrane was treated with a serum sample and then dipped into the chitosan–gold nanoparticle-GA-Protein A conjugate for detection [90].

By introducing an enhanced immuno-dot-blot assay, Safarpour et al. (2021) [90] significantly contributed to CE diagnosis (Figure 5). An anti-Ag B antibody was used with a gold nanoprobe and chitosan nanoparticle protein A to form a sandwich complex involving Ag B and chitosan–gold nanoparticle protein A. As a result of meticulously designed strategies, protein A was conjugated to gold nanoparticles, and Ag B was immobilized on nitrocellulose membranes. Simplicity and the ability to detect positive signals visually without complex equipment make this assay a useful diagnostic tool.

It is often challenging to diagnose cystic echinococcosis based on clinical symptoms and scanning. Establishing a definite diagnosis requires sensitive and reliable serological tests. In a recent study, silver nanoparticles were tested for their ability to detect circulating hydatid antigens in human serum samples using ELISAs [91]. Serum samples were collected from 66 people, including 36 with confirmed CE, 15 with parasites other than CE, and 15 without parasites. ELISA, nanosilver sandwich ELISA, and traditional methods were used to detect protoscolice antigens in circulating blood. According to the study, the nanosilver dot ELISA was 97.2% sensitive and 93.3% specific. Nanosilver sandwich ELISA had 94.4% and 96.7% sensitivity and specificity. Nano-silver-based ELISAs are more sensitive, specific, prognosticatory, and accurate than traditional ELISAs. Therefore, they are suitable for confirming cystic echinococcosis.

Moreover, several researchers studied 42 individuals with cystic echinococcosis and a control group [92]. By ELISA sandwich and Nanomagnetic Bead (NMB)-sandwich ELISA, anti-*E. granulosus* AgB-immunoglobulin (Ig) G polyclonal antibodies were prepared from human hydatid cysts. AgB was detected by sandwich ELISA with a high sensitivity and specificity of 88.9% and 91.7%, respectively, whereas NMB-sandwich ELISA saw AgB with 94.4% and 95.8%. NMB-sandwich ELISA was more accurate when measuring AgB in serum samples (95.2%), while urine samples revealed a slightly lower accuracy of 92.9%. 

## 3. Future and Prospective of Nanobiosensors

A nanobiosensor delivers extraordinary levels of sensitivity and specificity by using nanomaterials and biomolecular recognition elements [93]. A considerable advantage when dealing with CE detection is mainly if the parasite harbored into the intermediate host is at an early stage, the immunological response is still negative, and clinical signs are often lacking [94]. Diagnostic accuracy can be strongly improved by detecting nanobiosensors that identify antigens or genetic material associated with *E. granulosus s.l*. This enhanced sensitivity is crucial since it can handle subclinical infections and low parasite burdens that conventional diagnostic approaches cannot bear [95].

With nanobiosensors, real-time disease monitoring is possible, bringing a paradigm shift to the temporal dimension of disease management [96]. Conventional diagnostic methods take a long time to detect disease progression, while nanobiosensors catch it almost instantly [97]. Regarding CE, the feature is a game changer, as timely intervention can mitigate the severity of the infection. Changing disease management narratives is possible when healthcare providers make informed decisions.

Using nanobiosensors for point-of-care diagnostics marks a turning point [98]. Easily transportable, these devices reduce healthcare access disparities in remote or resource-limited settings [40]. Early detection is catalyzed by the availability of diagnostic tools on-site in endemic areas [99]. Additionally, decentralization minimizes the burden on centralized healthcare facilities, making medical care more affordable and efficient [100].

Detecting hydatid cysts simultaneously is now possible thanks to nanobiosensors’ intrinsic capability to accommodate multiplexing, enabling highly accurate diagnostics [25,64]. With this functionality, healthcare providers gain a holistic overview of diseases. A complex disease can be understood by detecting multiple antigens, parasite genotypes, or other pathogens [101]. 

By customizing nanobiosensors to fit the needs of environmental surveillance, diagnostics can extend beyond host detection [99]. Detecting *E. granulosus* eggs or antigens in the environment, such as soil, water, fruits, or vegetables, is part of an environmental surveillance program [102]. A deeper understanding of the parasite’s environmental reservoir sheds light on transmission dynamics. Directing control strategies more appropriately is possible by determining high-risk areas [103].

Integrating nanobiosensors with wireless technology and telemetry systems paves the way for new eras in disease surveillance [104]. Data are transmitted in real-time from nanobiosensors implanted or worn to a centralized repository [102]. Such dynamic remote monitoring makes it possible to continuously track infection dynamics and environmental influences. The application becomes particularly relevant when access to healthcare infrastructure is limited, facilitating the detection of infectious diseases and prompting action [105].

Hydatid cysts with nanobiosensors can be detected noninvasively or minimally invasively [25]. Hydatid cysts can be detected using nanoscale imaging technologies without invasive procedures in host tissues or bodily fluids [106]. Thus, not only is patient comfort enhanced, but procedural complications are minimized as well. Therefore, screening and monitoring programs are more likely to be complied with by patients.

Advanced data analytics and Artificial Intelligence (AI) algorithms are required to interpret the voluminous and intricate data generated by nanobiosensors [107]. A computer program can identify subtle patterns in data, predict disease trends, and make better decisions [108]. By identifying complex relationships within data, AI can unlock the diagnostic potential of nanobiosensors in surveillance programs [109].

Nanobiosensors are versatile, allowing researchers to tailor them to target specific biomarkers or accommodate regional variation in parasite genotypes [107]. By customizing the sensors, researchers can maximize their performance and ensure they are helpful in various epidemiological contexts [110]. Nanobiosensors can be calibrated precisely for different strains of *E. granulosus s.l.*, improving their suitability for other geographic areas [25].

Bioinformatics and genomic sequencing integrate harmoniously to provide a powerful genomic surveillance platform. In this way, genetic diversity in *E. granulosus s.l.* can be monitored, a crucial component of predicting disease dynamics and designing targeted control measures. Nanobiosensors, by analyzing genomic data, provide insights into parasite genotype distribution and disease transmission [111].

A collaborative approach is necessary to maximize nanosensors’ potential for detecting hydatid cysts. Innovation requires interdisciplinary collaboration among nanotechnologists, biologists, epidemiologists, and healthcare professionals. As technology advances, teamwork plays a vital role in uncovering new insights.

Biosensors generate data that allows epidemiologists to construct intricate maps and models of disease. These tools can visualize the geographic distribution of CE, identify high-risk regions, and optimize resource allocation. Based on insights generated from disease mapping, control strategies and efforts must be adapted [90].

## 4. Discussion

Despite its importance to global health, hydatid disease remains difficult to control without accurate and accessible diagnostic tools [90]. Nanotechnologies and nanobiosensors have advanced diagnostic capabilities in recent years. While these technologies have potential, challenges persist in harnessing them for CE diagnosis. With nanotechnology, which can manipulate materials at the nanoscale, diagnostic precision can be enhanced significantly [40]. Hydatid disease is prevalent in regions without access to cutting-edge technologies. Developing and implementing nanobiosensors may be costly for some settings with limited resources. This challenge can be overcome through cost-effective nanotechnology solutions that are easily integrated into different healthcare systems. Providing rapid and immediate diagnostic results with nanobiosensors is a beacon of hope for regions without laboratory facilities. In remote and underserved areas, disease epidemiology poses unique challenges to a point-of-care diagnosis of CE. Nanobiosensors are specifically designed to meet these needs [112]. In addition to being highly sensitive and specific, nanobiosensors must be robust enough to withstand different environments, which is a challenge for researchers [113]. A One Health approach is necessary for CE, a zoonotic disease affecting human and animal hosts [114]. The development of nanobiosensors has the potential to bridge the gap between human and veterinary diagnostics, enabling more integrated surveillance and control strategies. Fostering collaboration between human health and veterinary medicine is a challenging sector since they are traditionally distinct. To overcome health problems affecting humans and animals, interdisciplinary research and collaborative policy frameworks are essential. Several strains and genotypes of *E. granulosus* contribute to the disease complexity [88]. The molecular precision of nanobiosensors offers the possibility of detecting and differentiating between different strains of Echinococcus. It is crucial to develop nanobiosensors capable of detecting subtle genetic variations, and these special features have been reported in the medical oncology field [115]. Developing high-specificity nanobiosensors and understanding the parasite’s genetic landscape is essential [116]. For CE control programs to be effective, robust surveillance systems must exist. The potential for remote monitoring and real-time analysis of nanobiosensors can enhance disease surveillance significantly [117]. However, it remains challenging to integrate these technologies into existing surveillance frameworks. Aside from data management and interoperability, sensitive health data must be collected and shared ethically. Cystic echinococcosis surveillance will be strengthened if nanobiosensors can overcome these challenges [118]. Several socioeconomic factors influence the prevalence and persistence of hydatidosis. Economic constraints and cultural contexts must be considered when developing nanotechnological solutions. Educating the public and engaging the community is also necessary to address socioeconomic factors. Nanobiosensors should be technologically advanced and culturally and economically sensitive [119]. The distribution of *E. granulosus* and its hosts can be affected by environmental factors, including climate change [120,121]. Adaptable nanobiosensors allow monitoring and understanding of CE epidemiology as it changes over time. Adaptive surveillance strategies must integrate nanobiosensors into adaptive surveillance strategies while ensuring their continued effectiveness under shifting climate conditions [122]. In the context of CE control, *E. granulosus* is a potential source of antimicrobial resistance. In addition to informing treatment strategies, nanobiosensors can contribute to the sustainable use of anthelmintic drugs by monitoring drug resistance patterns. Identifying and integrating resistance markers into treatment decisions will be a significant challenge for nanobiosensors. Several challenges must be overcome before nanotechnology, and nanobiosensors can significantly advance the diagnosis of CE. Collaboration, innovative technology, and addressing community needs are essential for hydatid disease management [123]. By addressing these challenges head-on, scientists can contribute to more effective global control and prevention strategies, leading to breakthroughs in hydatidosis diagnosis. In veterinary medicine, nanobiosensors have also found applications and still hold significant potential for further development and utilization [124]. 

## 5. Conclusions

This study evidenced key insights into the immunological mechanisms of echinococcosis due to hydatid infection, highlighting the evolving understanding of immune responses triggered by hydatid infection. Clinical diagnosis of cystic echinococcosis with serum from intermediate hosts is a non-invasive and highly sensitive technique for identifying antibodies induced by *E. granulosus* antigens. Technological advancements, like nanobiosensors, would improve cystic hydatid treatment efficiency and enable early disease detection. Hydatid cyst biomarkers can now be detected more accurately and significantly more quickly with these nano-biosensors than with conventional biosensors. They offer higher sensitivity and versatility than conventional biosensors for detecting specific biomarkers. In addition, interpreting serological results requires understanding cross-reactivity and considering the disease stage. Generally, the improved integration of innovative diagnostic approaches, such as nanobiosensors, has led to significant advances in detecting and diagnosing cystic echinococcosis. This has contributed to better outcomes for patients and improvement in treatment efficacy.

## Data Availability

No further data were created or analyzed in this study. Authors are available to share those related to the manuscript.
